# Strontium-doping promotes bone bonding of titanium implants in osteoporotic microenvironment

**DOI:** 10.3389/fbioe.2022.1011482

**Published:** 2022-09-15

**Authors:** Tengyu Geng, Yiru Wang, Kaili Lin, Cheng Zhang, Jing Wang, Ya Liu, Changyong Yuan, Penglai Wang

**Affiliations:** ^1^ School of Stomatology, Xuzhou Medical University, Xuzhou, China; ^2^ Department of Dental Implant, The Affiliated Stomatological Hospital of Xuzhou Medical University, Xuzhou, China; ^3^ Department of Oral & Cranio-Maxillofacial Surgery, Shanghai Ninth People’s Hospital, Shanghai Jiao Tong University School of Medicine, Shanghai Key Laboratory of Stomatology, Shanghai Research Institute of Stomatology, Shanghai, China

**Keywords:** titanium implants, surface modification, osteoporosis, strontium, osseointegration

## Abstract

Osteoporosis is a major challenge to oral implants, and this study focused on improving the osseointegration ability of titanium (Ti) implants in osteoporosis environment *via* surface modification, including doping of strontium ion and preparation of nanoscale surface feature. Our previous studies have shown that strontium (Sr) ions can enhance osteogenic activity. Therefore, we aimed to comprehensively evaluate the effect of hydrothermal treatment of Sr-doped titanium implant coating on bone-binding properties in the microenvironment of osteoporosis in this study. We fabricated Sr-doped nanocoating (AHT-Sr) onto the surface of titanium implants *via* hydrothermal reaction. The rough Sr-doping had good biological functions and could apparently promote osteogenic differentiation of osteoporotic bone marrow mesenchymal stem cells (OVX-BMSCs). Most importantly, AHT-Sr significantly promoted bone integration in the osteoporosis environment. This study provides an effective approach to implant surface modification for better osseointegration in an osteoporotic environment.

## Introduction

Titanium (Ti) implants have been extensively utilized in the field of oral implant based on their good mechanical properties, bone-binding ability, and biocompatibility (Huang et al., 2004; Spriano et al., 2018). However, some factors may increase the risk of implant failure such as osteoporosis, diabetes, cancer, and smoking (Holahan et al., 2008; Chen et al., 2016; de Oliveira et al., 2020; Naseri et al., 2020; Zhang et al., 2021; Wei et al., 2022). Osteoporosis is a systemic bone metabolic disease caused by an imbalance between osteogenesis and osteoclast, which is often accompanied by a significant decrease in bone mineral density (Park et al., 2014; Takahashi et al., 2016; Russow et al., 2018). Osteoporosis can result in inadequate bone-implant contact, severely disrupting initial implant stability and bone integration (Du et al., 2016; Wang et al., 2021c). Osteoporosis is a challenge to the success of oral implants. There are significant changes of hormones and cytokines in bone marrows for patients with osteoporosis (Du et al., 2016). The pathological environment disrupts differentiation, proliferation, and intercellular communication of bone marrow stem cells in osteoporosis patients (Alghamdi et al., 2013; Dudeck et al., 2014). Therefore, titanium implants need to be biologically active to undergo bone integration in osteoporosis environment.

Several studies have shown the positive effects of strontium (Sr), tantalum, gallium, and zinc on osteogenic differentiation (Lin et al., 2013; Bonifacio et al., 2017; Zhao et al., 2019; Wang et al., 2021d). Sr is an important component of bone. It plays a key role in bone integration by promoting osteoblast differentiation and accelerating bone formation, while inhibiting osteoclast differentiation and reducing bone resorption (Choudhary et al., 2007; Bonnelye et al., 2008; Montagna et al., 2020). Sr-containing drugs play an active role in clinical application for anti-osteoporosis. However, oral Sr-containing drugs have low utilization rate and can cause systemic adverse reactions (Kolodziejska et al., 2021). Extensive efforts have focused on the construction of Sr-containing implant coatings for long-term and stable release. of Sr (Xing et al., 2020; Kuo et al., 2022). Bone marrow-derived mesenchymal stem cells (BMSCs), the precursors of osteoblastic-lineage cells, play a central role in bone formation (Bianco et al., 2011; Fu et al., 2022). Previous studies have demonstrated that Sr can promote MSCs differentiation in an normal physiological environment (Wang et al., 2020). However, the effect and underlying mechanism of strontium ion on MSCs in an osteoporotic environment remains unknown. Alkali heat treatment is a simple and stable method to construct Sr-containing coating on the surface of titanium implants, which leads to formation of nano-scale surface structures (Wang et al., 2018; Wang et al., 2020; Okuzu et al., 2021). Nanoscale surface features increase protein adsorption, stimulate osteoblast migration, and accelerate integration of bone and implants (Ding et al., 2020; Shu et al., 2020; Wang et al., 2022). By adjusting reaction conditions, the synergy of the two strategies provide a new idea for promoting osteoporotic bone bonding.

In this study, Sr-doped nanocoating was constructed on the implant surface by alkali heat treatment. BMSCs from osteoporotic rats were isolated and cultured and their biological properties, such as cell proliferation, cell morphology, and osteogenic differentiation, were estimated after AHT-Sr treatment. In addition, the osseointegration ability of titanium implants containing Sr-doped coating was evaluated *in vivo* in osteoporosis rats.

## Materials and methods

### Preparation of the materials

Titanium plates with dimensions of 10 mm × 10 mm × 1 mm and cylindrical implants consisting of titanium (2 mm × 4 mm) were employed in *vitro* and *in vivo* studies, respectively. The samples were polished to 2,000 grit with SiC sandpaper and cleaned by ultrasonic with acetone, ethanol, and deionized water. The samples were immersed in 5 M NaOH solution and subjected to hydrothermal treatment at 80°C for 6 h to form a rough surface, thoroughly ultrasonically cleaned in deionized water, and then wet-oxidized in deionized water at 200°C for 4 h. After thorough ultrasonic cleaning in deionized water, the samples were immersed in deionized water and 0.04 M SrCl_2_. The samples before alkali heat treatment (AHT) were labeled as Ti. The obtained samples treated with water and SrCl_2_ were labeled AHT and AHT-Sr, respectively.

### Surface characterization

The surface topography of the three groups of Ti plates was examined using field-emission scanning electron microscopy (FE-SEM; FEI Teneo VS, United States). X-ray energy-dispersive spectrometry (EDS; S4800, Hitachi) was used to observe the elemental composition above the three surfaces. Contact angle measurement (JY-82B Kruss DSA, Germany) was used to study and measure the wetting properties of the surface. The amount of Sr^2+^ leached was assessed by inductively coupled plasma atomic emission spectroscopy (ICP-MS; PerkinElmer NexION 300X, United States). The samples were placed in 10 ml of phosphate buffered saline (PBS) solution at 37°C and without stirring for various durations (1, 2, 3, 5, 7, 14 21, and 28 days).

### Cell culture

Forty 12-week-old female Sprague Dawley rats (average weight: 250 g) were used in this study. Thirty rats were randomly selected for bilateral ovariectomies surgery (OVX) to obtain osteoporotic condition. The remaining 10 rats were assigned to the control group and subjected to sham surgery. Twelve weeks later, the femurs of the two groups were taken for micro-CT to verify whether the model was successfully constructed. Meanwhile, BMSCs obtained from healthy rats (H-BMSCs) and OVX rats (OVX-BMSCs) were collected from the bone marrow of the tibia as well as femora of two rat groups as previously described. *In vitro* experiments were performed using cells from the third to fifth passages. H-BMCSs were seeded on Ti surfaces (Ctrl) while OVX-BMSCs were seed onto different Ti disk surfaces (Ti, AHT, and AHT-Sr). Animal experiments were performed under the authorization of the ethical committee of Xuzhou Medical University.

### Cell proliferation

BMSCs at a cell density of about 2 × 10^4^ cells/ml were seeded onto titanium surfaces and then cultured for 1, 4, or 7 days. At each time point, cell proliferation was assessed by the CCK-8 assay. After rinsing thrice with PBS, the cells were incubated in 400 μl of fresh culture medium (supplemented with 10% CCK-8 solution) at 37°C in the dark. After 2 h, 100 μl/well of the supernatant was transferred to a 96-well plate and absorbance at a wavelength of 450 mm was determined using a microplate reader (Thermo Fisher Scientific, Waltham, MA, United States).

### Initial cell adhesion

The BMSCs at a cell density of ∼2 × 10^4^ cells/ml were seeded onto titanium surfaces for 24 h. At various time points, the cells were washed thrice with PBS and then fixed with 4% PFA for 10 min at room temperature (RT). The samples were permeabilized using 0.1% Triton™ X-100 in PBS for 15 min. The samples were then stained with FITC-phalloidin and with DAPI for another 5 min in the dark following the manufacturer’s instructions. Cytoskeletal F-actin (red fluorescence) and cell nuclei (blue fluorescence) were assessed under an inverted fluorescence microscope (Olympus, IX73, Japan).

### Cell migration

BMSCs (cell density: 5,000 cells/well) were seeded onto the Ti substrate for 24 h, and a straight wound was made on the cell layer using a 1-ml pipette tip. After culturing for another 24 h, the cells were fixed with 4% PFA and stained with FITC-phalloidin and DAPI, then observed under fluorescence optics. Cell migration capacity was evaluated by measuring the width of the cell wound.

### Alkaline phosphatase staining

BMCSs were seeded at a density of about 2 × 10^4^ cells/ml and cultured in osteogenic induction media after adhering supplemented with 100 μg/ml ascorbic acid, 2 mmol/L β-glycerophosphate, and 10 nmol/L dexamethasone. Alkaline phosphatase (ALP) staining was conducted using a BCIP/NBT ALP kit (Beyotime, Shanghai, China) on days 4 and 7 of the cell culture following the manufacturer’s instructions.

### Quantitative Real-time PCR

BMSCs were seeded onto each sample at a density of 2 × 10^5^ cells/well. After culturing for 4 and 7 days, total RNA was extracted using TRIzol reagent (Takara Bio, Japan). A PrimeScript RT reagent kit (Takara Bio, Shiga, Japan) was used in reverse transcription of total RNA to cDNA. Real-time RT-PCR was conducted using a Quantitative SYBR Green Kit (Takara Bio, Shiga, Japan) and was detected by LightCycler480 System (Roche Diagnostics, Rotkreuz, Switzerland). Table 1 shows the primer sequences. The PCR conditions were as follows: 1) initial denaturation at 95°C for 30 s; 2) PCR: 95°C, 5 s; 60°C, 20 s, for 40 cycles; 3) melting: 95°C, 5 s; 60°C, 1 min; 95°C, for 1 cycle; and 4) cooling: 50°C, 30 s for 1 cycle. β-actin was used as control. The results were calculated using the 2^−ΔΔCT^ method.

**TABLE 1:** Primers used in qRT-PCR.

RNA	Sequence, 5′-3′
β-actin	Forward: GTA​AAG​ACC​TCT​ATG​CCA​ACA
	Reverse: GGA​CTC​ATC​GTA​CTC​CTG​CT
ALP	Forward: TAT​GTC​TGG​AAC​CGC​ACT​GAA​C
	Reverse: CAC​TAG​CAA​GAA​GAA​GCC​TTT​GG
BMP-2	Forward: GAA​GCC​AGG​TGT​CTC​CAA​GAG
	Reverse: GTG​GAT​GTC​CTT​TAC​CGT​CGT
BSP	Forward: AGA​AAG​AGC​AGC​ACG​GTT​GAG​T
	Reverse: GAC​CCT​CGT​AGC​CTT​CAT​AGC​C
COL-I	Forward: GCC​TCC​CAG​AAC​ATC​ACC​TA
	Reverse: GCA​GGG​ACT​TCT​TGA​GGT​TG
OCN	Forward: GCC​CTG​ACT​GCA​TTC​TGC​CTC​T
	Reverse: TCA​CCA​CCT​TAC​TGC​CCT​CCT​G
OPN	Forward: CCA​AGC​GTG​GAA​ACA​CAC​AGC​C
	Reverse: GGC​TTT​GGA​ACT​CGC​CTG​ACT​G

### Animal implant surgery

Twelve weeks post bilateral ovariectomy, the 20 OVX Sprague Dawley rats were randomly assigned to two groups (10 each), namely, AHT implants and AHT-Sr implants. After inducing general anesthesia using 10% sterile chloral hydrate solution, we made 1.8-mm-diameter implant holes in the femoral metaphysis (approximately 7 mm above the knee joint). A single implant was randomly placed into each femur. At 2 and 4 weeks after surgery, five rats in each group were sacrificed. We collected the femora and removed adherent tissues, and fixed these in 4% paraformaldehyde at RT for subsequent analysis.

### Micro-CT analysis of femora

The rats were sacrificed, and their femora were fixed with 4% phosphate buffered PFA. The samples were scanned using a SCANCO μCT 100 system (SCANCO Medical AG, Brüttisellen, Zurich, Switzerland) at a 4-μm resolution, 160 μA tube current, and 50 kV tube voltage. Regions of interest were selected within the 0.5–4.5 mm area below the growth plate at the distal ends of each femur.

### Statistical analysis

Data were expressed as the mean ± standard deviation (SD). Statistical analysis was performed using the IBM SPSS ver. 18.0. We assessed statistical differences using the two-way analysis of variance. Differences with a *p* < 0.05 were considered statistically significant.

## Results

### Characterization of Sr-doped nanocoating surface

**FIGURE 1:**
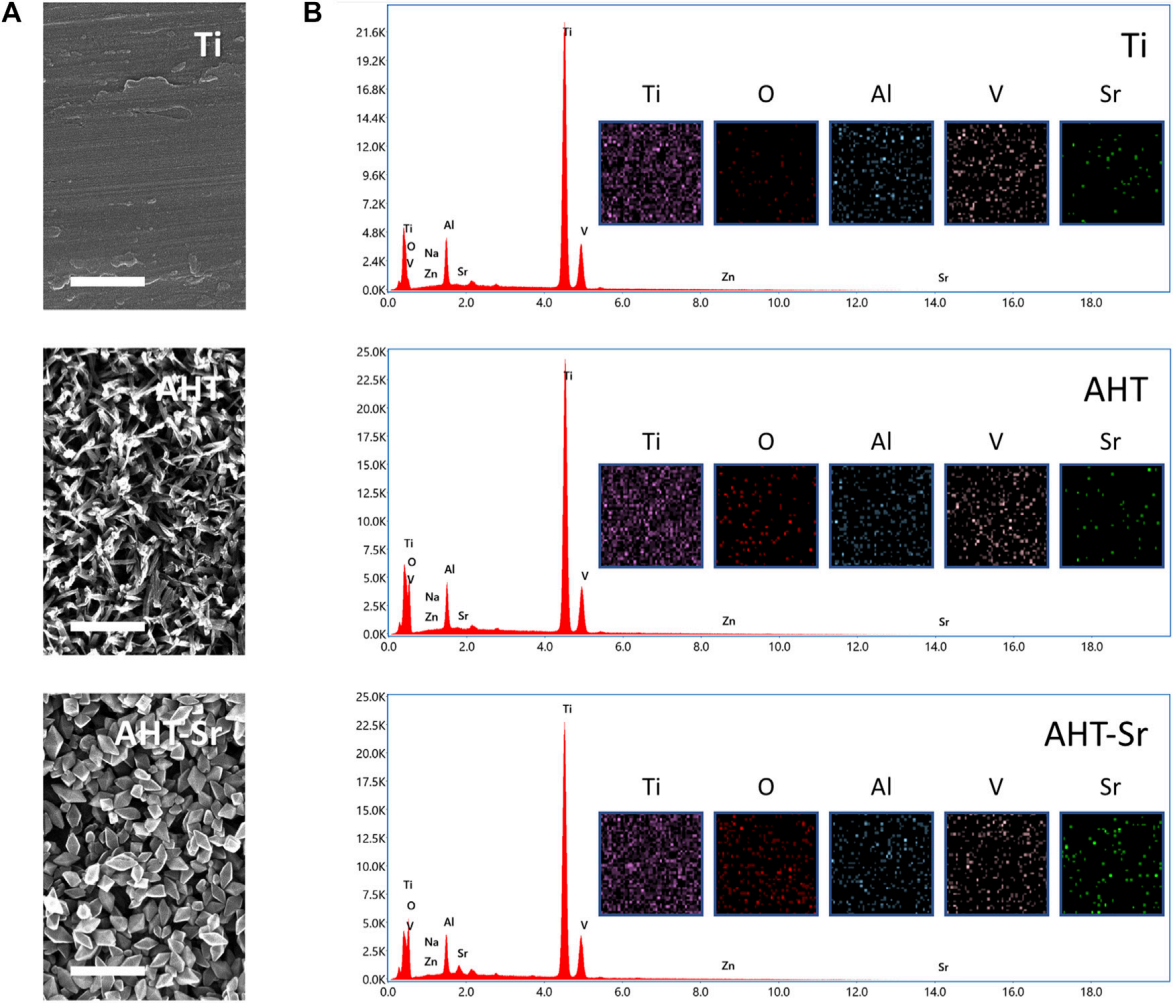
Surface characterizations of Ti, AHT, and AHT-Sr. **(A)** Representative SEM images showing the rough surface of Ti, AHT, and AHT-Sr. Scale bars: 500 nm. **(B)** EDS spectra and mappings of Ti, AHT, and AHT-Sr substrates.

Figure 1A Shows the SEM micrographs of samples with different coating parameters. The surface of Ti groups showed obvious polishing scratches. The AHT groups were characterized with a sponge network structure that was similar to our previous findings. Evenly distributed nanoscale (Ø 200–300 nm) particles could be found on AHT-Sr specimens. The EDS spectrum of AHT-Sr is shown in Figure 1B. The main elements of the AHT-Sr coating included Ti, O, Al, V, and Sr. Relative to the other two groups, Sr content increased in AHT-Sr, indicating that we had successfully Sr-doped the coating. The elemental composition of various samples as determined by EDS are shown in Table 2.

**TABLE 2:** Element contents on the surface of Ti, AHT, and AHT-Sr samples determined by EDS.

	Ti (wt%)	O (wt%)	Al (wt%)	V (wt%)	Sr (wt%)
Ti	86.3	1.5	5.7	4.9	0.6
AHT	73.5	16.2	4.4	4.4	0.6
AHT-Sr	71.8	17.6	4.0	3.7	1.9

**FIGURE 2:**
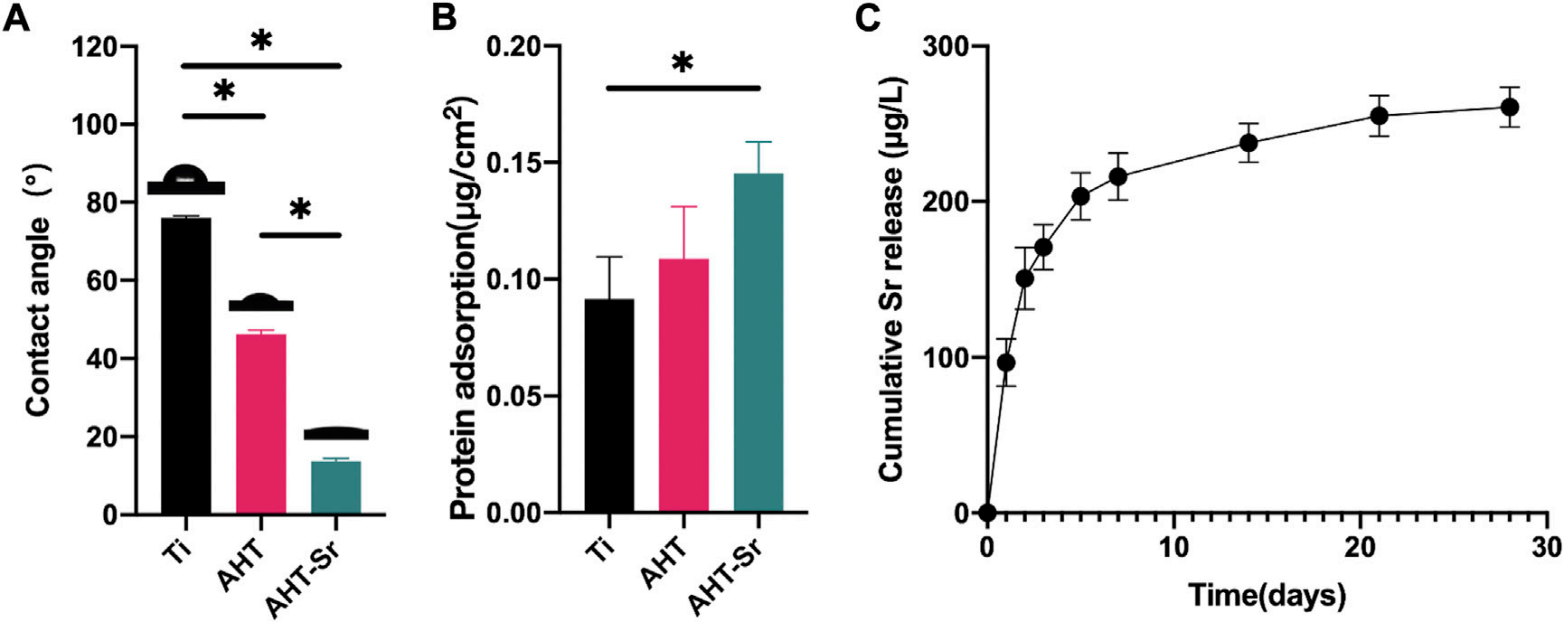
Surface physicochemical properties of Ti, AHT, and AHT-Sr. **(A)** Measurement of water contact angles in various specimens (*n* = 3). **(B)** Protein adsorption assay. **(C)** Cumulative release profile of Sr^2+^ ions from AHT-Sr within 28 days after incubation in PBS. The results are presented as the mean ± SD, *n* = 3, *p < 0.05.

In addition, the hydrophilic/hydrophobic ability of multiple samples were evaluated by water contact angle measurement (Figure 2A). The contact angle on the Ti and AHT was around 76.05° ± 0.47° and 46.32° ± 1.00°, respectively. However, the contact angle was 13.77° ± 0.73° on the AHT-Sr, indicating a high degree of hydrophilicity. This phenomenon may be attributable to the increase in surface roughness. Previous studies have indicated that osseointegration is influenced by the response of osteoblast cells to surface roughness and alteration in wettability. Protein adsorption test showed that AHT-Sr surface adsorbed more protein than Ti surface (Figure 2B). Figure 2C shows Sr ion release from AHT-Sr for 28 days. The accumulation concentration over the full 28 days was 260 μg/L.

### Establishment of osteoporotic conditions

**FIGURE 3:**
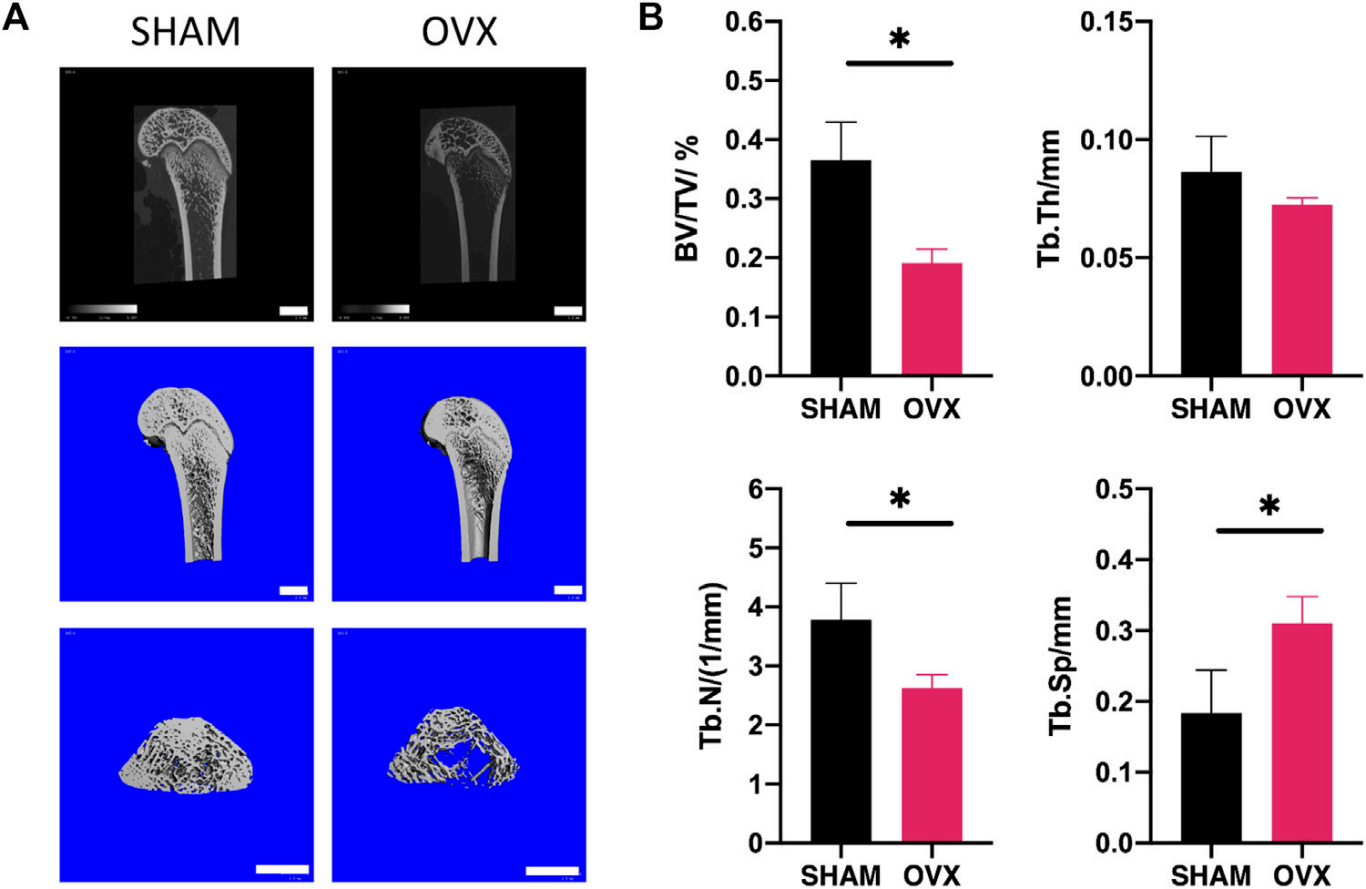
Establishment of OVX rat model for osteoporosis. OVX was performed at 12 weeks using micro-CT scanning to validate bone conditions. **(A)** 3D representative micro-CT micrographs of femoral condyles in the OVX and sham groups. Scale bars: 2 mm. **(B)** Quantitative parameters used to assess morphological bone alterations such as trabecular bone volume percentage (BV/TV), trabecular thickness (Tb.Th), trabecular number (Tp.N) and trabecular separation (Tb.Sp) (*n* = 6). Asterisks represent significant differences among groups (*p < 0.05).

OVX, a time-honored model to obtain osteoporotic condition, was used in this study. Here, bone conditions were evaluated by animal micro-computed tomography after 12 weeks of post-surgery to confirm whether our *in vivo* model of osteoporosis was established after OVX. Three-dimensional reconstruction showed a decline in bone level after OVX (Figure 3A). Twelve weeks after OVX, the volume of new bone and the thickness and number of trabecular bones significantly decreased, while the trabecular space significantly increased (Figure 3B).

### Cell attachment and proliferation on Titanium, AHT, and Sr-doped nanocoating samples

**FIGURE 4:**
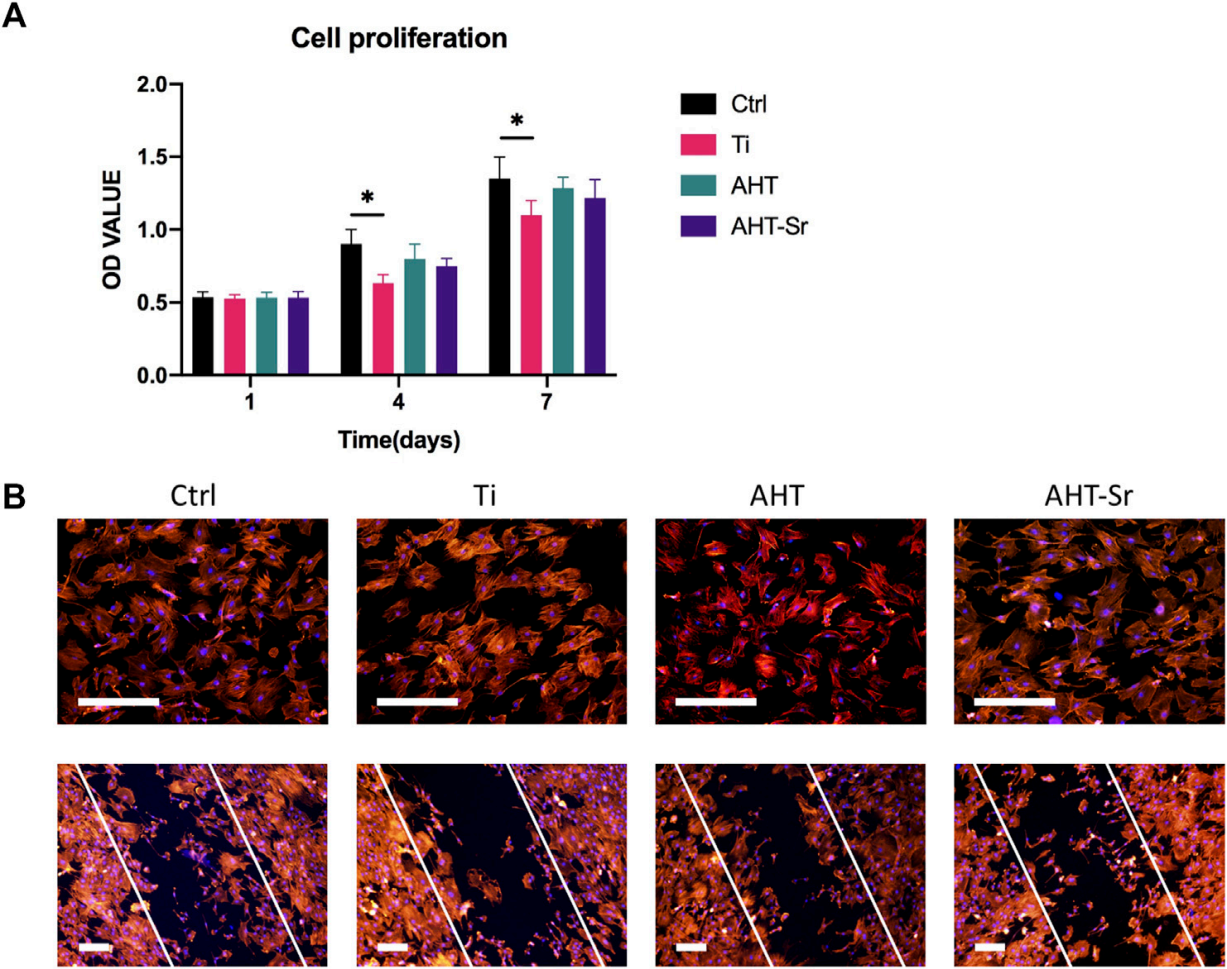
Biocompatibility studies on Ti disks. **(A)** Proliferation of H-BMSCs grown on Ti surfaces and OVX-BMSCs grown on different Ti disk surfaces. **(B)** F-actin immunostaining images of H-BMSCs and OVX-BMSCs grown on Ti, AHT, and AHT-Sr. Representative images of three separate experiments. Scale bars: 100 μm. Error bars indicate the SD of three separate experiments.

With cell proliferation, the number of OVX-BMSCs steadily increased over time. Similar proliferation rates of cells cultured on Ti, AHT, and AHT-Sr surfaces were observed for 7 days (Figure 4A). A scratch experiment was performed to assess MSC migration capacity (Figure 4B). After 12 h, the wound closure condition of OVX-BMSCs grown on an AHT or AHT-Sr surface was better compared to the Ti surface. Immunofluorescence staining of F-actin and nuclei were performed to evaluate initial cell attachment 4 h after cell seeding. OVX-BMSCs grown on the AHT and AHT-Sr surface showed clear filopodia extensions (Figure 4B). However, no significant differences in the initial adherent cell number on the Ti, AHT, and AHT-Sr samples were observed.

### Sr-doped nanocoating promotes osteogenic differentiation of osteoporotic bone marrow mesenchymal stem cells osteogenic differentiation in *vitro*

**FIGURE 5:**
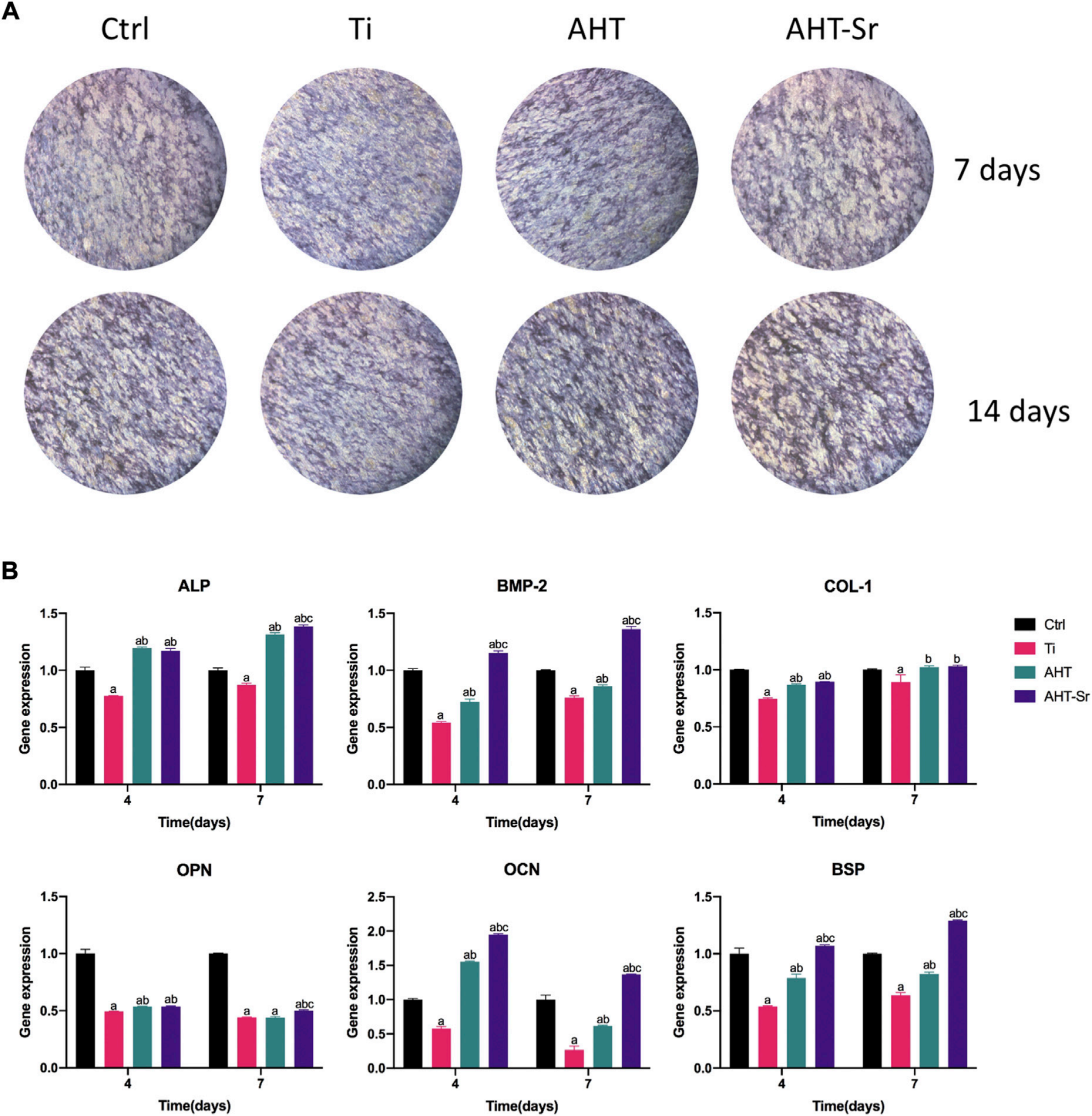
AHT-Sr promoted osteogenic differentiation of OVX-BMSCs. **(A)** ALP staining of H-BMSCs and OVX-BMSCs cultured on Ti, AHT and AHT-Sr disks for 4 and 7 days. Representative images of three separate experiments. **(Β)** mRNA expression of selected osteogenic markers in H-BMSCs and OVX-BMSCs that were cultured on different Ti disks for 4 and 7 days. Error bars indicate the SD of three separate experiment. *p < 0.05.

ALP activity and osteogenic gene expression of OVX-BMSCs were assessed to investigate osteogenic differentiation of OVX-BMSCs *in vitro*. First, ALP activity was detected by ALP staining after 4 and 7 days as a representative marker of early stage of osteogenic differentiation. According to Figure 5A, the cells cultured on the AHT-Sr surface showed darker purple dye effect compared to those cultured on the AHT coatings. Second, we assessed the expression of osteogenic markers using RT-PCT after culturing for four and 7 days. Compared with our findings from OVX-BMSCs cultured on AHT, the OVX-BMSCs grown on AHT-Sr exhibited upregulated ALP, BMP-2, COL-1, OCN, OPN, and BSP expression (Figure 5B). These results suggest that AHT-Sr, with released Sr^2+^ ions, facilitates early osteogenesis of OVX-BMSCs.

### Sr-doped nanocoating implant osseointegration in *vivo*

**FIGURE 6:**
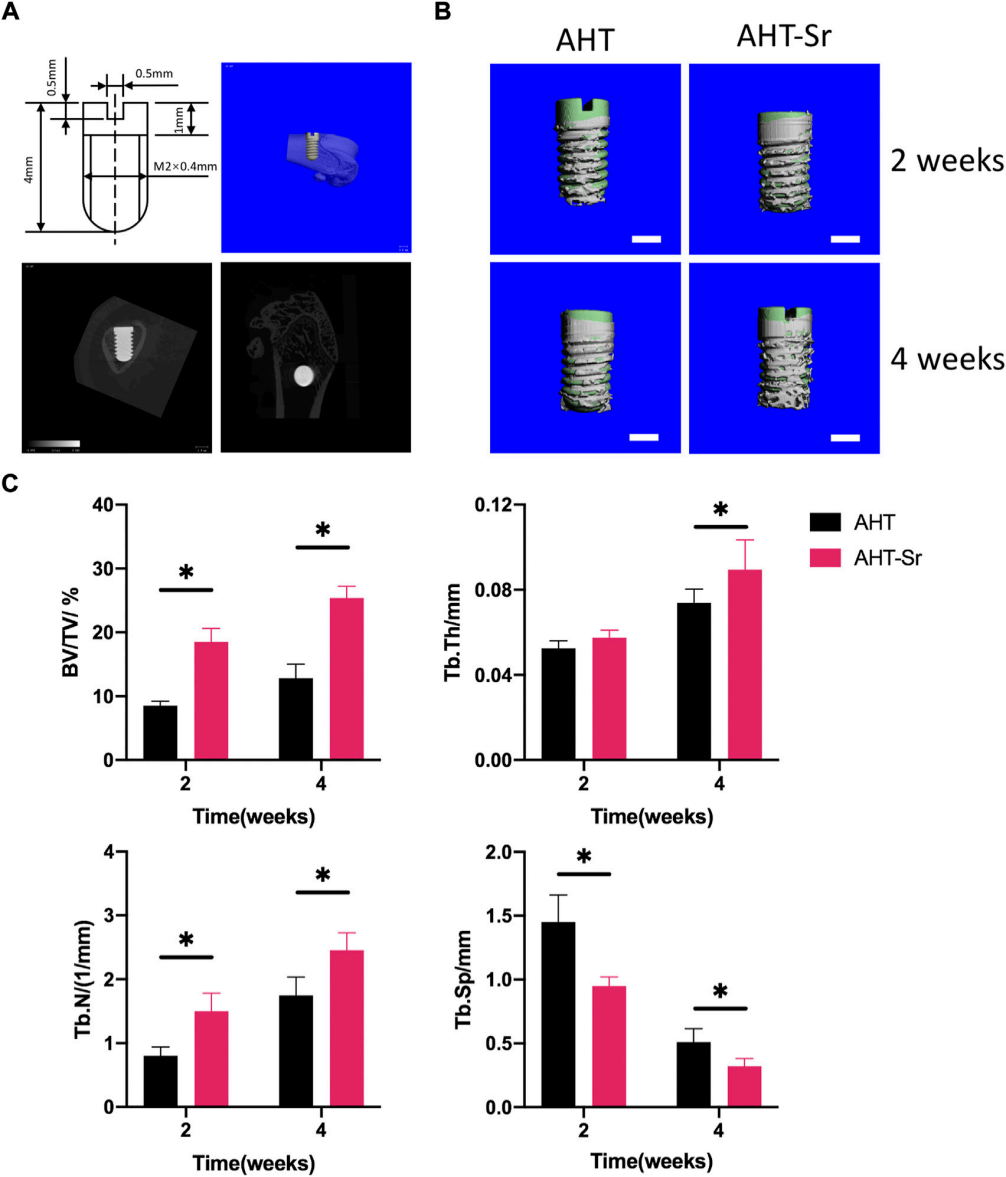
Osseointegration of AHT and AHT-Sr implants was measured by micro-CT. **(A)** Schematic of the surgery. **(B)** Reconstructed pictures of AHT implants (Control) and AHT-Sr implants at 2 and 4 weeks post surgery. Scale bar representing 1 mm; *n* = 5 per group. **(C)** BV/TV, Tb.Th, Tb.N, and Tb. Sp were measured. *n* = 5. *p < 0.05.

Previous studies have shown that AHT implants can enhance their osteogenic activity compared to Ti implants with smooth surface (Zhang et al., 2020; Wang et al., 2021). And treated implants with hydrophilic surfaces have been widely used in clinic. Therefore, we selected AHT as the control group *in vivo* experiments. Micro-CT was performed to analyze primary bone healing. Figure 6A shows the schematic diagram of 3D reconstruction including associated parameters. Significantly higher osteogenesis of AHT-Sr implants was observed relative to AHT implants after 2 and 4 weeks (Figure 6B). The volume of the newly formed bone and thickness and number of trabecular bones around the AHT-Sr implant were significantly higher than the AHT implants while trabecular space was significantly lower (Figure 6C).

## Discussion

Dysfunctional BMSCs result in bone formation defects in osteoporosis that in turn may cause implant failure (Du et al., 2016). In previous studies, coating with Sr ions on the implant surface can achieve better osteogenesis effect in health model (Zhao et al., 2019). However, the quantitative effect of AHT-Sr coatings on the osteoporosis model remains unclear. It would be of great significance to comprehensively evaluate the osseointegration following AHT-Sr implantation *in vivo*. Therefore, in this study, we successfully established an osteoporosis rat model. In addition, we coated nanoscale Sr onto AHT surfaces and found that in the osteoporosis model, AHT-Sr exhibited great osteogenic activity both *in vitro* and *in vivo*.

OVX rat models have been used in many studies for postmenopausal osteoporosis (Tao et al., 2015; Tao et al., 2016). In the present study, the trabecular bone volume of OVX rats markedly decreased in the distal femur 12 weeks after surgery, and BV/TV calculated by Micro-CT was significantly lower, which indicates that an osteoporosis model had been successfully established. Compared with H-BMSCs, VOX-BMSCs had decreased osteogenic differentiation ability and were used in *in vitro* studies.

As stated in the *in vitro* tests, OVX-BMSCs on AHT and AHT-Sr exhibited highly branched pseudopods extending into the microstructures of the coating (Figure 5). This result may be related to the roughness and good wettability of AHT-Sr surface. Previous studies have shown that that Ti implants with rough surfaces enhance osteoblast adhesion and extension compared to those with smooth surfaces (Gittens et al., 2014; Salou et al., 2015). The micro/nanotopography structures generated by hydrothermal treatment in this study were similar to those described in other studies (Kim et al., 2016). These micro/nanoscale structures observed through SEM on the AHT-Sr implant surface may enhance bone formation. In addition, osteoblast attachment could be promoted by implants with higher surface hydrophilicity (Le et al., 2021). Kubo et al. (2009) have reported that uniformly distributed 300-nm nano surface structures significantly enhance ALP activity, mRNA expression of Col1, and Ocn, and total calcium deposition in BMSCs. After alkali heat treatment, AHT-Sr induced VOX-BMSC differentiation *in vitro* with microstructures of approximately 300 nm in size that were randomly distributed. Surface wettability also influences implant osseointegration. Wilson et al. have shown that hydrophilic surfaces improve binding of adhesive proteins onto the osteoblast surface and promote their growth (Wilson et al., 2005; Deng et al., 2010). Moreover, greater surface wettability can speed healing and early bone bonding. We assumed an alike increase in osteogenic activity as wettability gradually increased from Ti and AHT to AHT-Sr (Figure 2).

As expected, the AHT-Sr had better osseointegration than the AHT considering Sr release. The chemical composition of coating is an important factor affecting the adhesion and attachment of osteoblasts (Wang et al., 2020). The release of Sr ions can alter local pH, increase cell microenvironment basicity, modify cell transmembrane protein structure, and improve the binding of cells onto proteins adsorbed on the AHT-Sr surface to promote adhesion (Zhang et al., 2014; Zhang et al., 2016; Schmidt et al., 2020). Over the years, in various experimental studies and clinical trials, a large number of studies have shown that stable Sr ions can promote bone formation and reduce bone resorption (Alghamdi and Jansen, 2013; Zhang et al., 2021).

Clinically, early osteogenesis has a significant impact on the success of implants. The degree of early osteogenesis relies on the ability of osteoblasts to generate new bone, which is controlled by related genes and proteins. ALP, COL-1, BMP-2, OCN, OPN, and BSP are indicators of osteoblast differentiation and mineralization and thus were assessed in this study (Figure 5B). ALP and COL-1 genes are early markers of osteoblast differentiation (Ding et al., 2016; Sun et al., 2021). OPN can adjust cell adhesion, migration, and mineral deposition as a multifunctional extracellular matrix protein (He et al., 2018). BMP-2 has an important role in activate osteoblasts and promote bone formation (Wang et al., 2021). OCN influences late differentiation of osteoblasts, which mainly occurs in the mineralization stage (Wan et al., 2022; Yu et al., 2022). *In vitro* RT-PCR analysis showed that the expression levels of these genes in cells cultured on AHT-Sr were significantly higher than those cultured on AHT. In addition, compared with AHT, ALP protein secretion, which promotes the mineralization of collagen matrix, was enhanced both *in vitro* and *in vivo* (Figure 5A). These findings suggest that AHT-Sr coating induces osteogenesis *in vitro* and *in vivo* by simultaneously maintaining bone regeneration and disrupting bone resorption. However, despite its beneficial effects, the mechanism by which Sr ions induce bone formation remains unclear. Sr can activate the CaSR and NFATc/Wnt signaling pathways and regulate OPG/RANKL and other mechanisms (Cui et al., 2020). Therefore, Sr imparts different effects on osteoclasts and osteoblasts, thereby resulting in increased bone mass, bone strength, and bone structure. Furthermore, Sr also influences the ras-MAPK signaling pathway. Peng et al. (2009) have reported that Sr upregulates Runx2 transcription and increases phosphorylation levels in human bone mesenchymal stem cells *via* the Ras-MAPK pathway, resulting in the upregulation of OCN, COL2A, OPN, and other genes that in turn enhances osteoblast differentiation. Zhang et al. (2018) found that micro structures and Sr ions could synergistically promote osteogenic differentiation by activating ERK1/2 and p38 MAPK signaling pathway. However, the mechanism of Sr ion induced bone formation remains unclear, and further studies are needed to clarify its underlying molecular mechanism.

The impact of AHT-Sr on osseointegration was assessed by histological analysis of micro-CT images for its potential clinical application. The results of this investigation showed that AHT-Sr coatings enhanced implant osseointegration and improved implant trabecular microstructure, thereby enhancing early implant osseointegration.

## Conclusion

In this study, we simulated the osteoporotic environment *in vitro* by culturing BMSCs isolated from the bone marrow of OVX Sprague Dawley rats to evaluate the therapeutic effect of AHT-Sr. After alkali heat treatment, AHT-Sr exhibited a rough surface with low contact angle and steadily released Sr ions. Based on our results, we showed that Sr-incorporated surfaces treated *via* hydrothermal reactions enhanced osteogenesic differentiation and early bone osseointegration using osteoporotic models and thus may potentially be used as a surface modification method for implant surfaces.

## Data Availability

The raw data supporting the conclusion of this article will be made available by the authors, without undue reservation.

## Abbreviations

Ti, titanium; Sr, Strontium; BMSCs, Bone marrow-derived mesenchymal stem cells;AHT, alkali-heat treated titanium; FE‐SEM, field‐emission scanning electron microscopy; EDS, energy-dispersive spectrometry; ICP-MS, inductively coupled plasma atomic emission spectroscopy; PBS, phosphate buffered saline; OVX, ovariectomies surgery; RT, room temperature; ALP, Alkaline phosphatase; SD, standard deviation.

## References

[B1] Alghamdi H. S. Bosco R. van den Beucken J. J. Walboomers X. F. Jansen J. A. (2013). Osteogenicity of titanium implants coated with calcium phosphate or collagen type-I in osteoporotic rats. Biomaterials 34 (15), 3747–3757. 10.1016/j.biomaterials.2013.02.033 23465489

[B2] Alghamdi H. S. Jansen J. A. (2013). Bone regeneration associated with nontherapeutic and therapeutic surface coatings for dental implants in osteoporosis. Tissue Eng. Part B Rev. 19 (3), 233–253. 10.1089/ten.TEB.2012.0400 23088597

[B3] Bianco P. Sacchetti B. Riminucci M. (2011). Stem cells in skeletal physiology and endocrine diseases of bone. Endocr. Dev. 21, 91–101. 10.1159/000328138 21865758

[B4] Bonifacio M. A. Cometa S. Dicarlo M. Baruzzi F. de Candia S. Gloria A. (2017). Gallium-modified chitosan/poly(acrylic acid) bilayer coatings for improved titanium implant performances. Carbohydr. Polym. 166, 348–357. 10.1016/j.carbpol.2017.03.009 28385242

[B5] Bonnelye E. Chabadel A. Saltel F. Jurdic P. (2008). Dual effect of strontium ranelate: Stimulation of osteoblast differentiation and inhibition of osteoclast formation and resorption *in vitro* . Bone 42 (1), 129–138. 10.1016/j.bone.2007.08.043 17945546

[B6] Chen Y. Chen X. Y. Shen J. W. He F. M. Liu W. (2016). The characterization and osteogenic activity of nanostructured strontium-containing oxide layers on titanium surfaces. Int. J. Oral Maxillofac. Implants 31 (4), e102–e115. 10.11607/jomi.4415 27447164

[B7] Choudhary S. Halbout P. Alander C. Raisz L. Pilbeam C. (2007). Strontium ranelate promotes osteoblastic differentiation and mineralization of murine bone marrow stromal cells: Involvement of prostaglandins. J. Bone Min. Res. 22 (7), 1002–1010. 10.1359/jbmr.070321 17371157

[B8] Cui X. Zhang Y. Wang J. Huang C. Wang Y. Yang H. (2020). Strontium modulates osteogenic activity of bone cement composed of bioactive borosilicate glass particles by activating Wnt/β-catenin signaling pathway. Bioact. Mater. 5 (2), 334–347. 10.1016/j.bioactmat.2020.02.016 32206735 PMC7078288

[B9] de Oliveira P. Bonfante E. A. Bergamo E. T. P. de Souza S. L. S. Riella L. Torroni A. (2020). Obesity/metabolic syndrome and diabetes mellitus on peri-implantitis. Trends Endocrinol. Metabolism 31 (8), 596–610. 10.1016/j.tem.2020.05.005 32591106

[B10] Deng Y. Morrissey S. Gathergood N. Delort A. M. Husson P. Costa Gomes M. F. (2010). The presence of functional groups key for biodegradation in ionic liquids: Effect on gas solubility. ChemSusChem 3 (3), 377–385. 10.1002/cssc.200900241 20049767

[B11] Ding Q. Cui J. Shen H. He C. Wang X. Shen S. G. F. (2020). Advances of nanomaterial applications in oral and maxillofacial tissue regeneration and disease treatment. WIREs Nanomed Nanobiotechnol 13, e1669. 10.1002/wnan.1669 33090719

[B12] Ding Y. F. Li R. W. Nakai M. Majumdar T. Zhang D. H. Niinomi M. (2016). Osteoanabolic implant materials for orthopedic treatment. Adv. Healthc. Mater. 5 (14), 1740–1752. 10.1002/adhm.201600074 27113724

[B13] Du Z. Xiao Y. Hashimi S. Hamlet S. M. Ivanovski S. (2016). The effects of implant topography on osseointegration under estrogen deficiency induced osteoporotic conditions: Histomorphometric, transcriptional and ultrastructural analysis. Acta Biomater. 42, 351–363. 10.1016/j.actbio.2016.06.035 27375286

[B14] Dudeck J. Rehberg S. Bernhardt R. Schneiders W. Zierau O. Inderchand M. (2014). Increased bone remodelling around titanium implants coated with chondroitin sulfate in ovariectomized rats. Acta Biomater. 10 (6), 2855–2865. 10.1016/j.actbio.2014.01.034 24534718

[B15] Fu Z. Zhuang Y. Cui J. Sheng R. Tomás H. Rodrigues J. (2022). Development and challenges of cells-and materials-based tooth regeneration. Eng. Regen. 10.1016/j.engreg.2022.04.003

[B16] Gittens R. A. Scheideler L. Rupp F. Hyzy S. L. Geis-Gerstorfer J. Schwartz Z. (2014). A review on the wettability of dental implant surfaces II: Biological and clinical aspects. Acta Biomater. 10 (7), 2907–2918. 10.1016/j.actbio.2014.03.032 24709541 PMC4103435

[B17] He Y. Mu C. Shen X. Yuan Z. Liu J. Chen W. (2018). Peptide LL-37 coating on micro-structured titanium implants to facilitate bone formation *in vivo* via mesenchymal stem cell recruitment. Acta Biomater. 80, 412–424. 10.1016/j.actbio.2018.09.036 30266635

[B18] Holahan C. M. Koka S. Kennel K. A. Weaver A. L. Assad D. A. Regennitter F. J. (2008). Effect of osteoporotic status on the survival of titanium dental implants. Int. J. Oral Maxillofac. Implants 23 (5), 905–910. 19014161

[B19] Huang P. Zhang Y. Xu K. Han Y. (2004). Surface modification of titanium implant by microarc oxidation and hydrothermal treatment. J. Biomed. Mat. Res. 70B (2), 187–190. 10.1002/jbm.b.30009 15264299

[B20] Kim H. S. Kim Y. J. Jang J. H. Park J. W. (2016). Surface engineering of nanostructured titanium implants with bioactive ions. J. Dent. Res. 95 (5), 558–565. 10.1177/0022034516638026 26961491

[B21] Kołodziejska B. Stępień N. Kolmas J. (2021). The influence of strontium on bone tissue metabolism and its application in osteoporosis treatment. Ijms 22 (12), 6564. 10.3390/ijms22126564 34207344 PMC8235140

[B22] Kubo K. Tsukimura N. Iwasa F. Ueno T. Saruwatari L. Aita H. (2009). Cellular behavior on TiO2 nanonodular structures in a micro-to-nanoscale hierarchy model. Biomaterials 30 (29), 5319–5329. 10.1016/j.biomaterials.2009.06.021 19589591

[B23] Kuo Y. J. Chen C. H. Dash P. Lin Y. C. Hsu C. W. Shih S. J. (2022). Angiogenesis, osseointegration, and antibacterial applications of polyelectrolyte multilayer coatings incorporated with silver/strontium containing mesoporous bioactive glass on 316L stainless steel. Front. Bioeng. Biotechnol. 10, 818137. 10.3389/fbioe.2022.818137 35223788 PMC8879691

[B24] Le P. T. M. Shintani S. A. Takadama H. Ito M. Kakutani T. Kitagaki H. (2021). Bioactivation treatment with mixed acid and heat on titanium implants fabricated by selective laser melting enhances preosteoblast cell differentiation. Nanomaterials 11 (4), 987. 10.3390/nano11040987 33921268 PMC8069428

[B25] Lin K. Xia L. Li H. Jiang X. Pan H. Xu Y. (2013). Enhanced osteoporotic bone regeneration by strontium-substituted calcium silicate bioactive ceramics. Biomaterials 34 (38), 10028–10042. 10.1016/j.biomaterials.2013.09.056 24095251

[B26] Montagna G. Cristofaro F. Fassina L. Bruni G. Cucca L. Kochen A. (2020). An *in vivo* comparison study between strontium nanoparticles and rhBMP2. Front. Bioeng. Biotechnol. 8, 499. 10.3389/fbioe.2020.00499 32612980 PMC7308719

[B27] Naseri R. Yaghini J. Feizi A. (2020). Levels of smoking and dental implants failure: A systematic review and meta‐analysis. J. Clin. Periodontol. 47 (4), 518–528. 10.1111/jcpe.13257 31955453

[B28] Okuzu Y. Fujibayashi S. Yamaguchi S. Masamoto K. Otsuki B. Goto K. (2021). *In vitro* study of antibacterial and osteogenic activity of titanium metal releasing strontium and silver ions. J. Biomater. Appl. 35 (6), 670–680. 10.1177/0885328220959584 32954894

[B29] Park Y. S. Lee J. Y. Suh J. S. Jin Y. M. Yu Y. Kim H. Y. (2014). Selective osteogenesis by a synthetic mineral inducing peptide for the treatment of osteoporosis. Biomaterials 35 (37), 9747–9754. 10.1016/j.biomaterials.2014.08.007 25205451

[B30] Peng S. Zhou G. Luk K. D. Cheung K. M. Li Z. Lam W. M. (2009). Strontium promotes osteogenic differentiation of mesenchymal stem cells through the Ras/MAPK signaling pathway. Cell Physiol. Biochem. 23 (1-3), 165–174. 10.1159/000204105 19255511

[B31] Russow G. Jahn D. Appelt J. Märdian S. Tsitsilonis S. Keller J. (2018). Anabolic therapies in osteoporosis and bone regeneration. Ijms 20 (1), 83. 10.3390/ijms20010083 PMC6337474 30587780

[B32] Salou L. Hoornaert A. Louarn G. Layrolle P. (2015). Enhanced osseointegration of titanium implants with nanostructured surfaces: An experimental study in rabbits. Acta Biomater. 11, 494–502. 10.1016/j.actbio.2014.10.017 25449926

[B33] Schmidt R. Gebert A. Schumacher M. Hoffmann V. Voss A. Pilz S. (2020). Electrodeposition of Sr-substituted hydroxyapatite on low modulus beta-type Ti-45Nb and effect on *in vitro* Sr release and cell response. Mater. Sci. Eng. C 108, 110425. 10.1016/j.msec.2019.110425 31923935

[B34] Shu T. Zhang Y. Sun G. Pan Y. He G. Cheng Y. (2020). Enhanced osseointegration by the hierarchical micro-nano topography on selective laser melting Ti-6Al-4V dental implants. Front. Bioeng. Biotechnol. 8, 621601. 10.3389/fbioe.2020.621601 33490056 PMC7817818

[B35] Spriano S. Yamaguchi S. Baino F. Ferraris S. (2018). A critical review of multifunctional titanium surfaces: New frontiers for improving osseointegration and host response, avoiding bacteria contamination. Acta Biomater. 79, 1–22. 10.1016/j.actbio.2018.08.013 30121373

[B36] Sun Y. Li Y. Zhang Y. Wang T. Lin K. Liu J. (2021). A polydopamine-assisted strontium-substituted apatite coating for titanium promotes osteogenesis and angiogenesis via FAK/MAPK and PI3K/AKT signaling pathways. Mater. Sci. Eng. C 131, 112482. 10.1016/j.msec.2021.112482 34857268

[B37] Takahashi T. Watanabe T. Nakada H. Tanimoto Y. Kimoto S. Mijares D. Q. (2016). Effect of a dietary supplement on peri-implant bone strength in a rat model of osteoporosis. J. Prosthodont. Res. 60 (2), 131–137. 10.1016/j.jpor.2015.12.006 26787534 PMC4975944

[B38] Tao Z. S. Lv Y. X. Cui W. Huang Z. L. Tu K. K. Zhou Q. (2016). Effect of teriparatide on repair of femoral metaphyseal defect in ovariectomized rats. Z Gerontol. Geriat 49 (5), 423–428. 10.1007/s00391-015-0949-1 26358080

[B39] Tao Z. S. Zhou W. S. Tu K. K. Huang Z. L. Zhou Q. Sun T. (2015). Effect exerted by Teriparatide upon Repair Function of β-tricalcium phosphate to ovariectomised rat's femoral metaphysis defect caused by osteoporosis. Injury 46 (11), 2134–2141. 10.1016/j.injury.2015.07.042 26306803

[B40] Wan H. Y. Shin R. L. Y. Chen J. C. H. Assunção M. Wang D. Nilsson S. K. (2022). Dextran sulfate-amplified extracellular matrix deposition promotes osteogenic differentiation of mesenchymal stem cells. Acta Biomater. 140, 163–177. 10.1016/j.actbio.2021.11.049 34875356

[B41] Wang A. Yuan W. Song Y. Zang Y. Yu Y. (2022). Osseointegration effect of micro-nano implants loaded with kaempferol in osteoporotic rats. Front. Bioeng. Biotechnol. 10, 842014. 10.3389/fbioe.2022.842014 35284417 PMC8905647

[B42] Wang H. Liu J. Wang C. Shen S. G. Wang X. Lin K. (2021a). The synergistic effect of 3D-printed microscale roughness surface and nanoscale feature on enhancing osteogenic differentiation and rapid osseointegration. J. Mater. Sci. Technol. 63 (04), 18–26. 10.1016/j.jmst.2019.12.030

[B43] Wang H. Xu Q. Hu H. Shi C. Lin Z. Jiang H. (2020). The fabrication and function of strontium-modified hierarchical micro/nano titanium implant. Ijn Vol. 15, 8983–8998. 10.2147/IJN.S268657 PMC7682802 33239873

[B44] Wang H. Zhang X. Wang H. Zhang J. Li J. Ruan C. (2018). Enhancing the osteogenic differentiation and rapid osseointegration of 3D printed Ti6Al4V implants via nano-topographic modification. J. Biomed. Nanotechnol. 14 (4), 707–715. 10.1166/jbn.2018.2551 31352944

[B45] Wang M. Li H. Yang Y. Yuan K. Zhou F. Liu H. (2021b). A 3D-bioprinted scaffold with doxycycline-controlled BMP2-expressing cells for inducing bone regeneration and inhibiting bacterial infection. Bioact. Mater. 6 (5), 1318–1329. 10.1016/j.bioactmat.2020.10.022 33210025 PMC7658329

[B46] Wang X. Li Z. Wang Z. Liu H. Cui Y. Liu Y. (2021c). Incorporation of bone morphogenetic protein-2 and osteoprotegerin in 3D-printed Ti6Al4V scaffolds enhances osseointegration under osteoporotic conditions. Front. Bioeng. Biotechnol. 9, 754205. 10.3389/fbioe.2021.754205 34805113 PMC8600075

[B47] Wang X. Ning B. Pei X. (2021d). Tantalum and its derivatives in orthopedic and dental implants: Osteogenesis and antibacterial properties. Colloids Surfaces B Biointerfaces 208, 112055. 10.1016/j.colsurfb.2021.112055 34438295

[B48] Wei H. Cui J. Lin K. Xie J. Wang X. (2022). Recent advances in smart stimuli-responsive biomaterials for bone therapeutics and regeneration. Bone Res. 10 (1), 17. 10.1038/s41413-021-00180-y 35197462 PMC8866424

[B49] Wilson C. J. Clegg R. E. Leavesley D. I. Pearcy M. J. (2005). Mediation of biomaterial-cell interactions by adsorbed proteins: A review. Tissue Eng. 11 (1-2), 1–18. 10.1089/ten.2005.11.1 15738657

[B50] Xing H. Li R. Wei Y. Ying B. Li D. Qin Y. (2020). Improved osteogenesis of selective-laser-melted titanium alloy by coating strontium-doped phosphate with high-efficiency air-plasma treatment. Front. Bioeng. Biotechnol. 8, 367. 10.3389/fbioe.2020.00367 32478042 PMC7235326

[B51] Yu X. Wang X. Li D. Sheng R. Qian Y. Zhu R. (2022). Mechanically reinforced injectable bioactive nanocomposite hydrogels for *in-situ* bone regeneration. Chem. Eng. J. 433, 132799. 10.1016/j.cej.2021.132799

[B52] Zhang C. Zhang T. Geng T. Wang X. Lin K. Wang P. (2021a). Dental implants loaded with bioactive agents promote osseointegration in osteoporosis: A review. Front. Bioeng. Biotechnol. 9, 591796. 10.3389/fbioe.2021.591796 33644012 PMC7903335

[B53] Zhang J. Liu J. Wang C. Chen F. Wang X. Lin K. (2020). A comparative study of the osteogenic performance between the hierarchical micro/submicro-textured 3D-printed Ti6Al4V surface and the SLA surface. Bioact. Mater. 5 (1), 9–16. 10.1016/j.bioactmat.2019.12.008 31956731 PMC6956677

[B54] Zhang J. Zhao S. Zhu Y. Huang Y. Zhu M. Tao C. (2014). Three-dimensional printing of strontium-containing mesoporous bioactive glass scaffolds for bone regeneration. Acta Biomater. 10 (5), 2269–2281. 10.1016/j.actbio.2014.01.001 24412143

[B55] Zhang W. Cao H. Zhang X. Li G. Chang Q. Zhao J. (2016). A strontium-incorporated nanoporous titanium implant surface for rapid osseointegration. Nanoscale 8 (9), 5291–5301. 10.1039/c5nr08580b 26881868

[B56] Zhang X. Cui J. Cheng L. Lin K. (2021b). Enhancement of osteoporotic bone regeneration by strontium-substituted 45S5 bioglass via time-dependent modulation of autophagy and the Akt/mTOR signaling pathway. J. Mat. Chem. B 9 (16), 3489–3501. 10.1039/d0tb02991b 33690737

[B57] Zhang X. Li H. Lin C. Ning C. Lin K. (2018). Synergetic topography and chemistry cues guiding osteogenic differentiation in bone marrow stromal cells through ERK1/2 and p38 MAPK signaling pathway. Biomater. Sci. 6 (2), 418–430. 10.1039/c7bm01044c 29340362

[B58] Zhao Q. Yi L. Jiang L. Ma Y. Lin H. Dong J. (2019). Surface functionalization of titanium with zinc/strontium-doped titanium dioxide microporous coating via microarc oxidation. Nanomedicine Nanotechnol. Biol. Med. 16, 149–161. 10.1016/j.nano.2018.12.006 30594657

